# Whole transcriptome analysis highlights nutrient limitation of nitrogen cycle bacteria in simulated microgravity

**DOI:** 10.1038/s41526-024-00345-z

**Published:** 2024-01-10

**Authors:** Tom Verbeelen, Celia Alvarez Fernandez, Thanh Huy Nguyen, Surya Gupta, Raf Aarts, Kevin Tabury, Baptiste Leroy, Ruddy Wattiez, Siegfried E. Vlaeminck, Natalie Leys, Ramon Ganigué, Felice Mastroleo

**Affiliations:** 1 Nuclear Medical Applications, Belgian Nuclear Research Centre (SCK CEN), Boeretang 200, 2400 Mol, Belgium; 2 Center for Microbial Ecology and Technology (CMET), Ghent University, Coupure Links 653, 9000 Ghent, Belgium; 3 Department of Proteomics and Microbiology, University of Mons, Av. Du Champs de Mars 6, 7000 Mons, Belgium; 4 Research Group of Sustainable Energy, Air and Water Technology, Department of Bioscience Engineering, University of Antwerp, Groenenborgerlaan 171, 2020 Antwerp, Belgium; 5 Centre for Advanced Process Technology for Urban REsource Recovery (CAPTURE), Frieda Saeysstraat 1, 9052 Ghent, Belgium

**Keywords:** Microbiology, Microbial ecology

## Abstract

Regenerative life support systems (RLSS) will play a vital role in achieving self-sufficiency during long-distance space travel. Urine conversion into a liquid nitrate-based fertilizer is a key process in most RLSS. This study describes the effects of simulated microgravity (SMG) on *Comamonas testosteroni*, *Nitrosomonas europaea*, *Nitrobacter winogradskyi* and a tripartite culture of the three, in the context of nitrogen recovery for the Micro-Ecological Life Support System Alternative (MELiSSA). Rotary cell culture systems (RCCS) and random positioning machines (RPM) were used as SMG analogues. The transcriptional responses of the cultures were elucidated. For CO_2_-producing *C. testosteroni* and the tripartite culture, a PermaLife^TM^ PL-70 cell culture bag mounted on an in-house 3D-printed holder was applied to eliminate air bubble formation during SMG cultivation. Gene expression changes indicated that the fluid dynamics in SMG caused nutrient and O_2_ limitation. Genes involved in urea hydrolysis and nitrification were minimally affected, while denitrification-related gene expression was increased. The findings highlight potential challenges for nitrogen recovery in space.

## Introduction

The capacity to supply food, water, and a breathable atmosphere in a robust and reliable manner over time is one of the major challenges in long-distance space travel beyond low Earth orbit (LEO). In that regard, regenerative life support systems (RLSS) have recently attracted significant attention due to their potential to allow circular resource recovery and utilization in the scope of long-duration space missions. Over 30 years ago, the European Space Agency initiated the Micro-Ecological Life Support System Alternative (MELiSSA)^1^. MELiSSA consists of five compartments populated by micro-organisms, higher plants or the crew. Its purpose is the total recovery of the elements or molecules in the crew’s waste streams, converting them to potable water, oxygen and food^2^. In the initial MELiSSA configuration, the third compartment (CIII) is responsible for the conversion of NH_4_^+^ to NO_3_^− 2^. Currently, NH_4_^+^ is oxidized aerobically to NO_2_^−^ by the autotrophic gram-negative NH_4_^+^-oxidizing bacterium *Nitrosomonas europaea*. *Nitrobacter winogradskyi*, an autotrophic gram-negative NO_2_^−^-oxidizing bacterium, converts NO_2_^−^ to NO_3_^−^ aerobically, which is then used as a nitrogen source for cyanobacteria and higher plants in compartments IVa (CIVa) and IVb (CIVb), respectively^2^. The initial nitrification system CIII is metabolically unable to directly treat urine. However, efficient urine treatment is necessary since urine contains 85% of recoverable N in a RLSS, mostly present as urea^3^. Urea can be hydrolyzed to NH_4_^+^ and CO_2_ in a process called ureolysis. A previous study already showed the feasibility of ureolysis in combination with nitrification using the heterotrophic gram-negative urease-positive bacterium *Comamonas testosteroni* in a synthetic tripartite community together with *N. europaea* and *N. winogradskyi*^4^. Furthermore, such a heterotroph is needed to convert the oxidizable organics contained in urine, enabling recovery of the majority of this carbon as CO_2_, and to avoid fueling undesired microbial growth in a downstream compartment.

In the context of a RLSS design for space, the microgravity environment might play an important role in activity of the N-cycle species. It reduces shear forces and minimizes convection, hydrostatic forces and density differences in fluid systems^5^. Consequently, a nutrient-depleted zone can form around metabolically active bacterial cells because the nutrients only disperse through the slow diffusion process^6,7^. Experimental work on bacteria in both real and simulated microgravity (SMG) has shown pronounced effects on the bacterial metabolism^8,9^, nutrient availability^10^, bacterial proliferation^9,11^, motility and biofilm formation^12–14^, quorum sensing^15^, osmolarity^14,16^, secondary metabolism^17^, stress resistance^10,16,18^ and virulence^9,17^.

The effects of microgravity on ureolytic and nitrifying communities still have to be elucidated. Previous spaceflight experiments have shown that *N. europaea* and *N. winogradskyi,* inactivated by storage in respectively NH_4_^+^ or NO_2_^−^-depleted growth medium, can be reactivated by addition of the nitrogen electron donor after exposure to spaceflight in LEO^19,20^. Active nitrification, on the other hand, has been performed in the Closed Equilibrated Biological Aquatic System (C.E.B.A.S.)^21,22^ and in a mission of the National Space Development Agency of Japan (NASDA)^23^. In these artificial aquatic ecosystems, a consortium of ammonium-oxidizing bacteria removed NH_4_^+^ excreted by fish to NO_3_^−^, and retained good water quality for the fish^21–23^. However, in situ ureolytic and nitrification activities in the context of RLSS design, and the effect of spaceflight on the responsible strains, have not been demonstrated yet. This paper represents terrestrial experiments conducted with microgravity analogs to understand the consequences of simulated microgravity (SMG) on gene expression of N-cycle bacteria.

SMG is mimicked with a rotary cell culture system (RCCS) or a random positioning machine (RPM), which respectively produce low-shear modeled microgravity (LSMMG) or randomized simulated microgravity (RSMG). The RCCS continuously rotates perpendicular to the gravity vector, keeping the cells in a suspended orbit. As a result, it creates a constant free-fall of the cells through the culture medium. Solid body rotation of the liquid is generated and fluid dynamics are minimized^7,24^. On the other hand, an RPM rotates with random accelerations and orientations in a 3D plane. This theoretically causes bacterial cells to experience a nullified net gravity vector^25,26^. Fluid motion in the RPM, however, is characterized by increased shear forces and enhanced convection as opposed to LSMMG and real microgravity^25^. Both microgravity-analog devices were used in this study to provide a more complete picture of the effects of different SMG conditions.

A global transcriptional analysis was performed on both axenic *C. testosteroni*, *N. europaea* or *N. winogradskyi* cultures as well as on the tripartite culture cultivated in SMG conditions. To our knowledge, this study is the first to analyze the effects of spaceflight-analog conditions on the gene expression profiles of nitrogen cycle bacteria.

## Results and discussion

### Fluid mixing in cell culture bags mounted on RCCS and RPM hardware

**Fig. 1:**
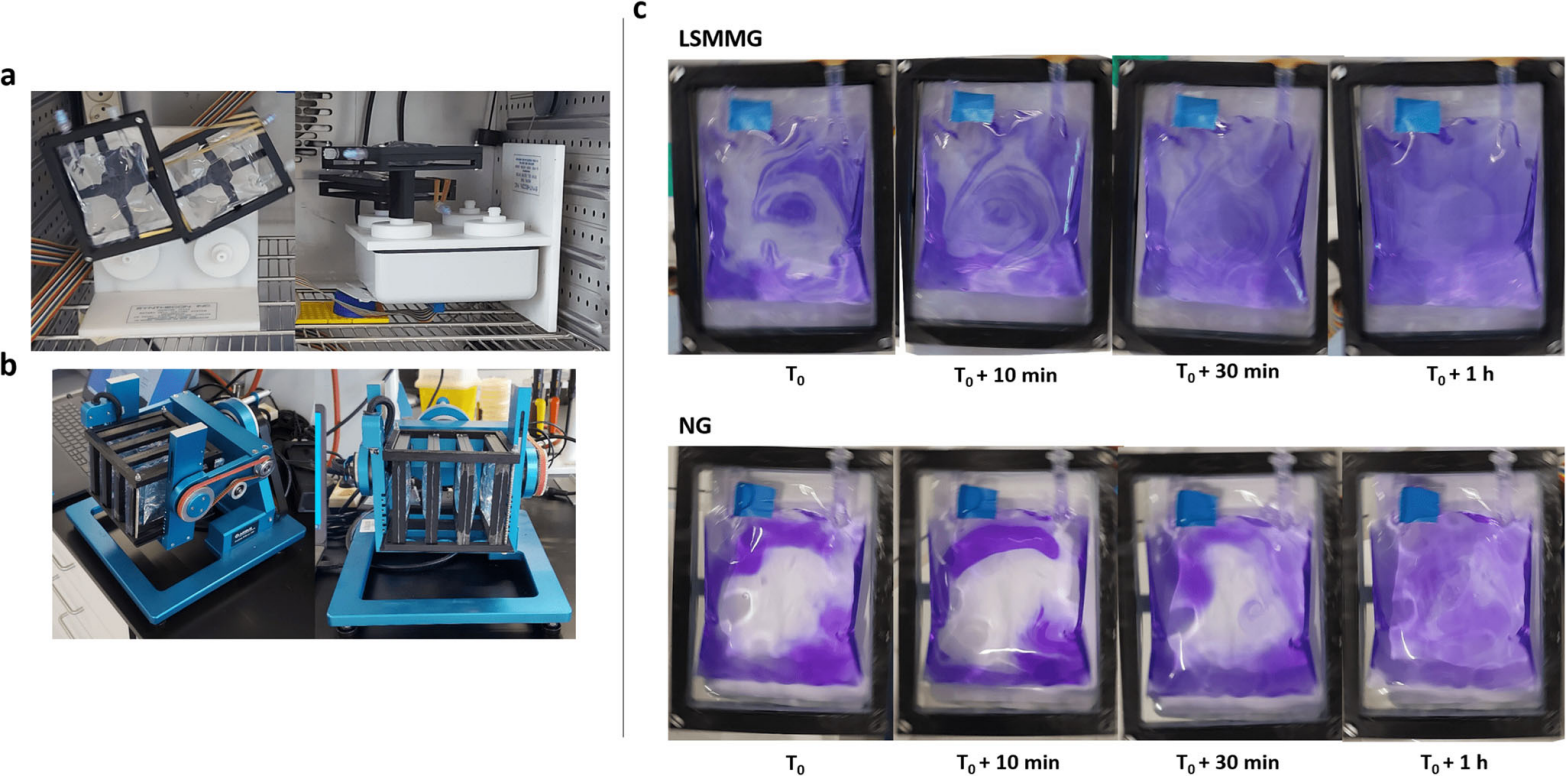
Simulated microgravity setup with PL-70 cell culture bags. Pictures of in-house designed 3D-printed cell culture bag holders for (**a**) Rotary cell culture system (RCCS) and (**b**) Random positioning machine (RPM) cultivation and (**c**) Mixing of crystal violet in normal gravity (NG) and low-shear modeled microgravity (LSMMG) after injection with a needle syringe through the sample port.

During the first cultivation of *C. testosteroni* and the tripartite culture in regular RCCS vessels, we noticed that the vessels were unable to eliminate gaseous CO_2_ produced during ureolysis and oxidation of organics through its gas-permeable membrane. These gas bubbles heavily disrupt the solid-body rotation of the liquid in LSMMG. Consequently, they strongly increase shear rate and introduce additional fluid dynamics that negate the SMG environment. For this reason, an alternative to the conventional RCCS container, that was used for both LSMMG and RSMG, was introduced. PermaLife^TM^ PL-70 cell culture bags are made of a gas-permeable fluoroethyl polymer film that ensures gas exchange with the environment, but with a larger surface area and possibly a higher gas-exchange rate than the gas-permeable membrane on the RCCS. Using in-house designed 3D-printed holders (Fig. 1a, b) for these cell culture bags, we were able to grow *C. testosteroni* and the tripartite community in LSMMG and RSMG with substantially less gas bubble formation. Throughout SMG cultivation, gas bubbles did not have to be removed, resulting in a continuous SMG environment without disruption to eliminate formed gas pockets.

The level of fluid dispersal in normal gravity (NG), LSMMG and RSMG in cell culture bags was assessed using a 0.03% crystal violet solution to validate their use for SMG experiments (Fig. 1c). In both NG and LSMMG, the crystal violet dispersed in a spiral-like pattern immediately after rotation started. After 10 min, 30 min and 1 h of rotation, slow mixing gradually occurred. After 2 h, the dye was completely mixed in both conditions (pictures after 2 h not shown).

In the conventional RCCS vessel, solid-body rotation takes place with minimal mixing occurring in LSMMG^10,13^. With the use of cell culture bags, on the other hand, the observed mixing is probably a residual effect stemming from the non-cylindrical shape of the bag. Near the corners, the liquid deviates from the circular trajectory and consequently affects the uniformity of the circular motion in the center of the liquid, adding small shear dynamics. During the growth of *C. testosteroni* in LSMMG, an aggregate of bacterial mass remained stationary during rotation, as shown in the video in the supplemental information (Supplemental Movie 1). This provided us with a visual confirmation that the biomass and potentially the fluid in close proximity to the biomass exhibited negligible movement and remained in a “free-fall” state during the rotational motion of the system. Meanwhile, fluid mixing in the RPM was almost instantaneous, as was also previously observed in another study by Crabbe et al.^10^. Within 10 s, the dye was homogeneously dispersed in the liquid. The fluid dynamics of the RPM in a cell culture bag should be highly similar to any other experiment conducted with the RPM, since any container filled to capacity can be considered in an experimental setup with dimensions suitable for the RPM. Due to their efficacy in mitigating air bubble formation during SMG studies on *C. testosteroni*, these PL cell bags can also be utilized for future SMG studies concerning gas-producing microorganisms. For example, *Limnospira indica* and other photosynthetic oxygenic microorganisms can produce consumable biomass and convert CO_2_ to O_2,_ producing a considerable amount of gas bubbles in the process. This makes them difficult to cultivate them in SMG. These organisms often form a crucial element within a RLSS and insights into their growth under SMG conditions will be very valuable for the development of these systems.

### Culture growth under simulated microgravity conditions

**Fig. 2:**
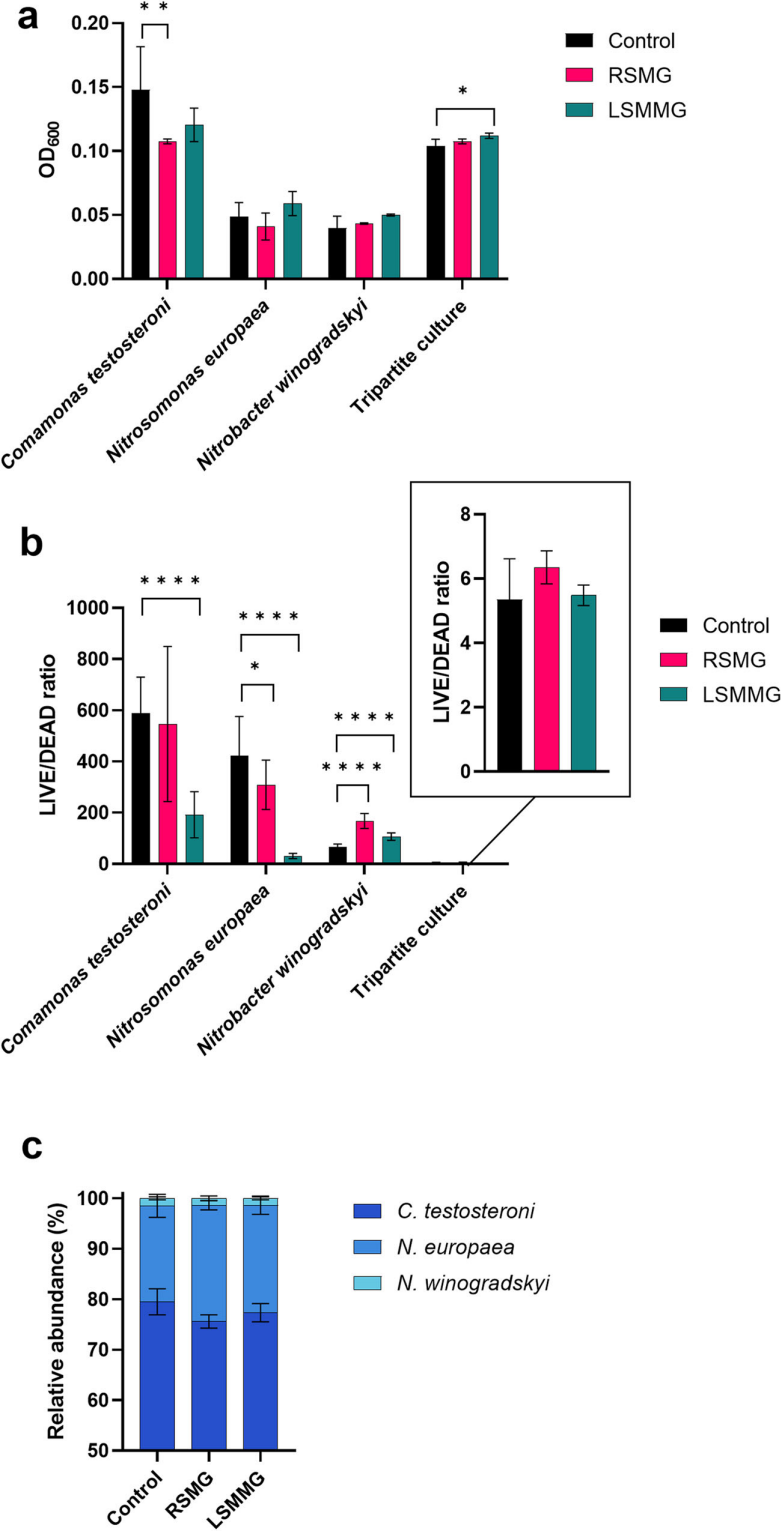
Growth of N-cycle bacteria in simulated microgravity (SMG). **a** Endpoint OD600 measurements, **b** LIVE/DEAD ratio of C*. testosteroni*, *N. europaea*, *N. winogradskyi* and the tripartite culture grown in randomized simulated microgravity (RSMG) and in low-shear modeled microgravity (LSMMG) conditions. **c** Relative abundance of *C. testosteroni*, *N. europaea* and *N. winogradskyi* in the synthetic tripartite community after 20 days of growth in SMG. Analysis of Variance (ANOVA) and post-hoc Tukey’s tests were performed to identify significant differences in the final cell density and LIVE/DEAD ratios between the two SMG conditions and the normal gravity (NG) control. *p < 0.05, **p < 0.01, ****p < 0.0001. Data represents the mean ± SD (*n* = 4).

OD_600_ measurements (Fig. 2a) and LIVE/DEAD ratios (Fig. 2b) of the cultures grown in RSMG, LSMMG and NG were determined at the experiment endpoint. This occurred after 3 days of growth in SMG for *C. testosteroni*, 5 days for the nitrifiers and 20 days for the tripartite culture. LIVE/DEAD ratios were calculated by dividing the total number of intact cells (LIVE) by the total number of damaged cells (DEAD) and was used as a viability indicator.

No significant differences in biomass were identified for the axenic nitrifying strains. *C. testosteroni* in RSMG grew significantly less biomass (ANOVA, *p* = 0.0059) in comparison to NG while measured cell density of the tripartite culture in LSMMG was significantly higher (ANOVA, *p* = 0.0255) compared to NG.

In previous SMG studies, increased growth was observed in several *Escherichia coli* strains, *Salmonella enterica, Salmonella enteriditis*, *Stenotrophomonas maltophilia* and *Vibrio natriegens* during growth in LSMMG or spaceflight conditions^27–33^, whereas *Rhodospirillum rubrum, Staphylococcus aureus* and *E. coli* K12 did not differ in proliferation in LSMMG^15,34,35^. The aerobic growth rate of *Lactobacillus acidophilus* was increased in LSMMG but the final cell density did not, while anaerobically grown *L. acidophilus* displayed no differences in growth rate compared to NG^36,37^. Finally, two studies on *Pseudomonas aeruginosa* or *Limosilactobacillus* (formerly *Lactobacillus*) *reuteri* in both RMSG and LSMMG displayed no proliferation differences compared to NG^10,18^. An array of other species also show either increased or similar growth in LSMMG (reviewed in refs. ^9,38^). To our knowledge, *C. testosteroni* is the only bacterium that displayed decreased cell densities in RSMG, which has not been observed previously in other RSMG-growing strains but is consistent with some spaceflight studies^29,30^.

LIVE/DEAD analysis identified a significant decline in viability (ANOVA, *p* < 0.0001) of axenic *C. testosteroni* in LSMMG and of axenic *N. europaea* in RSMG (ANOVA, *p* = 0.0285) and LSMMG (ANOVA, *p* < 0.0001) while the viability of *N. winogradskyi* was increased in both RSMG (ANOVA, *p* < 0.0001) and LSMMG (ANOVA, *p* < 0.0001). No differences were observed in the viability of the tripartite culture compared to NG, but the viability was sharply decreased in comparison to the axenic cultures. However, this might be a consequence of the culture’s age, reaching 20 days. At this stage in a batch configuration, *C. testosteroni* is already far into its stationary phase, which could have resulted in higher cellular turnover. In previous SMG experiments, *V. natriegens*^11^ and *L. reuteri*^18^ displayed a higher survival compared to NG while no differences in viability were observed in *Streptococcus mutans*^39^. The former observations are consistent with *N. winogradskyi*’s viability in SMG. On the other hand, we observed decreased viabilities of *C. testosteroni* and *N. europaea*, which was not found, to our knowledge, in other bacteria grown in SMG. Meanwhile, the viability of the tripartite culture in LSMMG was comparable to the NG control but the culture displayed an increased final cell density, indicating a potential positive effect on growth for all or some of the strains in LSMMG. How microgravity affects bacterial growth remains ambiguous and seems to be dependent on the bacterial species or strain^27–32^, growth conditions (such as oxygen availability and type of medium)^36,37^, and the method of simulating microgravity and the presence of other bacterial species, as observed in this study. Finally, similarities in proliferation and viability in SMG compared to NG do not necessarily reflect that the bacteria do not experience any effects from SMG. This was also observed in *S. mutans*, where similar proliferation and viabilities were observed accompanied by a significant shift in the transcriptomic and metabolomic profile when cultured in LSMMG^39^.

To determine differences in the relative abundance of the three strains present in the synthetic community, qPCR was conducted (Fig. 2c). *C. testosteroni* exhibited the highest biomass proportion in all three conditions, with an average abundancy of 80%, 76%, and 77% after 20 days of growth in respectively NG, RSMG and LSMMG. *N. europaea* accounted for 19%, 23%, and 21% while *N. winogradskyi* contributed 1.5%, 1.4% and 1.4% to the tripartite community. *N. europaea* and *N. winogradskyi* grew in a ratio of ~15:1 across all three conditions. In a previous chemostat co-culture study, the ammonia and nitrite oxidizers were present in a ratio of 4:1, while in a biofilm reactor, the relative abundance varied from 9:1 to 3:7 along the height of the bioreactor^40–42^. In the bioreactor, NH_4_^+^ at the inlet was consumed by a high population density of *N. europaea*. Due to high NH_4_^+^ concentrations, *N. europaea* in the lower regions was very active and likely outcompeted *N. winogradskyi* for O_2_, since *N. europaea* possesses a lower half saturation constant (*K*_*m*_) (1–15 µM O_2_) than *N. winogradskyi* (22–166 µM O_2_)^43^. Gradually, *N. winogradskyi* abundance increases due to heightened NO_2_^−^ availability and lower NH_4_^+^ levels through NH_3_ oxidation by *N. europaea*^41,42^. In the current study, it is possible that *N. europaea* outcompeted *N. winogradskyi* for all O_2_ in SMG and NG in a batch configuration, as suggested by Ilgrande et al.^4^. In their clinostat experiment (another type of SMG simulator closely related to RCCS microgravity simulation) of the tripartite culture, no NO_3_^−^ accumulation was observed in LSMMG nor NG, suggesting inactivity and inhibition of NO_2_^−^ oxidation of *N. winogradskyi* and, consequently, its growth^4^. The negative impact on *N. winogradskyi* growth in a tripartite context in batch culturing was also observed in this study. To our knowledge, no data exists on relative abundance in a tripartite ureolytic and nitrifying culture. In SMG, the relative distribution of N-cycle bacteria does not appear to be affected in the tripartite culture. The final cell density of the tripartite culture was significantly higher in LSMMG compared to NG, however. This suggests that all species increased their proliferation in the LSMMG scenario, possibly through a synergistic promotion of each other’s growth.

### Whole transcriptome analysis of nitrogen cycle bacteria in simulated microgravity conditions

#### The transcriptomic landscape of Comamonas testosteroni in SMG

**Fig. 3:**
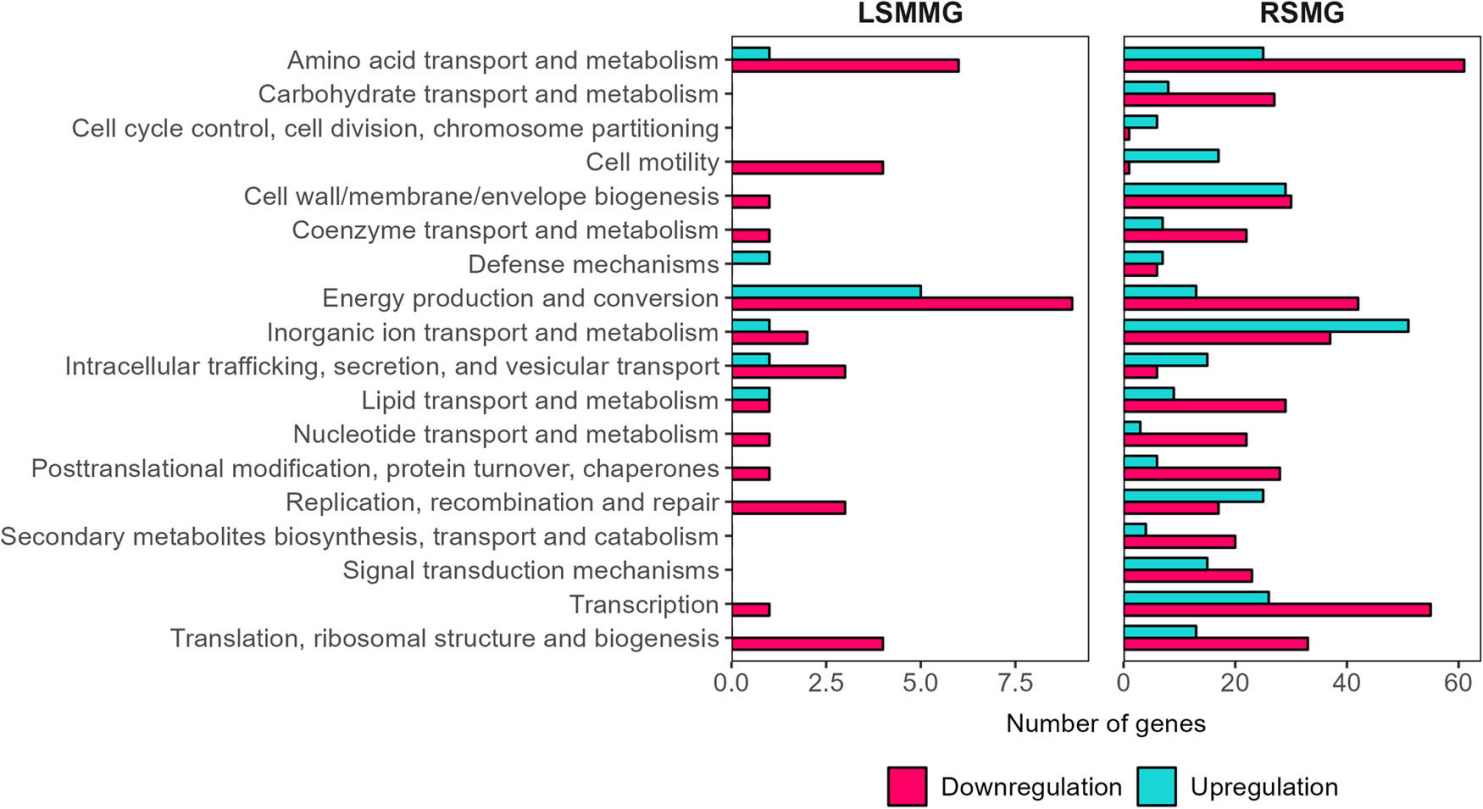
Differentially expressed genes per cluster of orthologues genes (COG) category of axenic *C. testosteroni* in simulated microgravity conditions. Number of genes that are differentially regulated in each COG category in axenic *C. testosteroni* grown in low-shear modeled microgravity (LSMMG) and randomized simulated microgravity (RSMG) conditions. COG categories ‘Function unknown’ and ‘General function prediction only’ were excluded.

In axenic *C. testosteroni* exposed to SMG conditions, the effect of RSMG was notably greater than the effect of LSMMG on the transcriptome compared to NG. We identified 1168 differentially expressed genes (DEG) ((*p* < 0.05) and −1 ≥ log_2_ fold change (FC) ≥ 1 or |FC | ≥ 2) of which 469 were upregulated and 699 were downregulated in RSMG. In contrast, only 71 DEGs in LSMMG were found, of which 20 genes were overexpressed and 51 genes were underexpressed (Supplementary Data 1). An overview of the number of DEGs per cluster of orthologous groups (COGs) is provided in Fig. 3. In the next sections, these DEGs and the implications of their differential expression will be discussed. From here on out, when discussing the ‘axenic’ strain, we refer to it within a monoculture context. On the other hand, when addressing the strain in the tripartite culture, we discuss that strain within the tripartite community.

‘Amino acid transport and metabolism’, ‘Energy production and conversion’, ‘Inorganic ion transport and metabolism’, and ‘Transcription’ COGs were most represented in RSMG. In LSMMG, on the other hand, only the ‘Amino acid transport and metabolism’ and ‘Energy production and conversion’ were abundantly represented. Respectively 24 and 429 DEGs were annotated to ‘Function unknown’ in LSMMG and RSMG. No genes were annotated to ‘General function prediction only’.

#### RSMG has a strong effect on the metal ion homeostasis of Comamonas testosteroni

Metal ion homeostasis was among the most affected cellular processes of axenic *C. testosteroni* in RSMG. An overview of the DEGs involved in these processes can be found in Table 1. Expression of three heavy metal efflux systems of the resistance nodulation division (RND) family was strongly repressed with a FC of −11.47 down to −40.50. Expression of one RND efflux pump was upregulated. Downregulation of heavy metal efflux pumps was previously observed in LSMMG experiments on *V. natriegens*^11^ and *E. coli*^44,45^. On the other hand, upregulation of some heavy metal transport proteins was observed in *Cupriavidus metallidurans* in RSMG^46^ and in *Burkholderia contaminans* and the yeast *Candida albicans* subjected to LSMMG or spaceflight, respectively^13,47^. These heavy metal transporters were mostly predicted to have a role in multidrug resistance^48^. Other than differential expression of heavy metal pump genes, increases in antibiotic resistance in some strains was observed after spaceflight or LSMMG exposure^36,47,49^. In this study, the observed downregulation of these genes in axenic *C. testosteroni* could suggest a reduction in multidrug resistance after proliferation in RSMG. However, no general consensus behind the mechanisms that result in either heightened or diminished multidrug resistance in SMG or spaceflight conditions has been established.

**Table 1:** Differentially expressed genes involved in metal ion homeostasis of axenic *Comamonas testosteroni* in simulated microgravity.

Gene ID	Gene name	Description	Fold change in RSMG	Fold change in LSMMG
METAL ION EXPORT				
JMRS01_360163	N/A	Efflux transporter, RND family, MFP subunit	−13.64	ND
JMRS01_360164	N/A	Heavy metal efflux system protein	−22.78	ND
JMRS01_360165	N/A	Outer membrane protein, heavy metal efflux system	−11.47	ND
JMRS01_860360	N/A	Heavy metal efflux system protein	−11.55	ND
JMRS01_860361	N/A	Membrane fusion protein, heavy metal efflux system	−23.10	ND
JMRS01_860362	N/A	RND efflux system, outer membrane lipoprotein, NodT family	−15.03	ND
JMRS01_1050029	N/A	Outer membrane protein, heavy metal efflux system	−19.84	ND
JMRS01_1050030	N/A	Heavy metal efflux system protein	−23.10	ND
JMRS01_1050031	N/A	Membrane fusion protein, heavy metal efflux system	−40.50	ND
JMRS01_320074	N/A	Outer membrane heavy metal efflux system	2.07	ND
JMRS01_600011	N/A	Efflux RND transporter periplasmic adapter subunit	2.77	−2.13
JMRS01_600012	N/A	Putative heavy metal efflux pump, CzcA family	2.71	ND
JMRS01_600013	N/A	Putative outer membrane efflux protein	3.41	ND
METAL ION IMPORT				
Molybdenum				
JMRS01_10023	*mopIII*	Molybdenum-pterin binding protein 3	2.93	ND
JMRS01_240055	*modB*	Molybdate transport system permease	2.22	ND
JMRS01_260193	*modB*	Molybdate transport system permease	2.28	ND
Magnesium				
JMRS01_360413	*corA*	Magnesium transport protein	−2.71	ND
JMRS01_600152	*mgtE*	Putative MgtE-domain containing protein	−7.16	ND
JMRS01_600153	*mgtA*	P-type Mg^2+^ transporter	−14.00	ND
JMRS01_600154	*mgtC*	Mg^2+^ transporter-C	−7.14	ND
JMRS01_1050028	*mgtA*	P-type Mg^2+^ transporter	−5.35	ND
Zinc				
JMRS01_10033	*gufA*	Zinc-iron permease	3.05	ND
Iron				
JMRS01_10040	*exbD*	Biopolymer transport protein ExbD1	2.20	ND
JMRS01_10048	*fiu*	Catecholate siderophore receptor	−7.21	ND
JMRS01_160014	*fecA*	Iron complex outer membrane receptor protein	3.68	ND
JMRS01_160015	*fes*	Putative Fe^3+^-enterobactin esterase	9.06	ND
JMRS01_200038	*fecA*	TonB-dependent receptor	−3.12	ND
JMRS01_240067	N/A	Iron complex transport system permease protein	3.03	2.23
JMRS01_400027	*hmuV*	Hemin import ATP-binding protein	16.11	ND
JMRS01_400028	*hmuU*	Hemin transport system permease	23.59	ND
JMRS01_400029	*hmuT*	Hemin transport substrate-binding protein	13.27	ND
JMRS01_400030	*hmuS*	Putative hemin transport protein	25.81	ND
JMRS01_400031	N/A	Conserved protein of unknown function	16.68	ND
JMRS01_400032	*hmuR*	Putative hemin receptor	6.77	ND
JMRS01_400036	*bfr*	Bacterioferritin	−2.95	ND
JMRS01_430016	*fecI*	RNA polymerase sigma-70 factor, ECF subfamily	2.57	ND
JMRS01_430017	*fecR*	Anti-FecI sigma factor, FecR	2.69	ND
JMRS01_430018	*fecA*	Putative TonB-dependent receptor	1.91	ND
JMRS01_510008	*fecA*	Outer membrane receptor for ferric coprogen	2.17	ND
JMRS01_560010	*fecA*	Fe^3+^ dicitrate transport protein FecA	−2.28	ND
JMRS01_560028	*bfr*	Bacterioferritin	−2.71	ND
JMRS01_600115	*fiu*	Iron catecholate outer membrane transporter Fiu	8.69	ND
JMRS01_600116	*ybiX*	PKHD-type hydrolase YbiX	8.57	ND
JMRS01_600118	*fecA*	Outer membrane receptor for ferrienterochelin and colicins	5.82	ND
JMRS01_600258	N/A	Iron complex transport system substrate-binding protein	12.55	ND
JMRS01_600259	*fecA*	Putative TonB-dependent receptor	11.88	ND
JMRS01_600260	N/A	Conserved protein of unknown function	10.85	ND

*ND* Not differentially expressed, *RSMG* Randomized simulated microgravity, *LSMMG* Low-shear simulated microgravity

Upregulation of a range of genes involved in metal ion import was also observed, while iron storage bacterioferritin- and Mg^2+^ import-coding genes were repressed. Fe-S cluster assembly protein-coding gene *cyaY* (JMRS01_700189), which slows down Fe-S assembly^50^, was also downregulated. Upregulation of iron and metal import genes implies iron or metal limitation for the bacteria^51^. Hence, it is possible that *C. testosteroni* grown in RSMG experienced iron limitation due to depletion of these resources in the vicinity of the cell. Moreover, through downregulation of *cyaY*, the bacteria seemed to promote Fe-S cluster assembly in RSMG, which is essential in various cellular processes.

In LSMMG, on the other hand, only two DEGs involved in metal ion homeostasis were identified. As such, it does not seem that axenic *C. testosteroni* is altering its metal ion homeostasis in this SMG condition.

#### Comamonas testosteroni experiences energy limitation in RSMG

The transcriptome of axenic *C. testosteroni* in RSMG suggests a deprivation of essential nutrients and O_2_. For one, 27 out of 33 identified DEGs involved in amino acid biosynthesis were repressed while transcript levels of six were increased (Table 2). Translation machinery was also generally inhibited, with expression of 43 out of 62 DEGs involved in ribosomal biogenesis, tRNA and aminoacyl-tRNA biosynthesis and other translation-related genes being downregulated. A SoxR-coding gene (*soxR*; JMRS01_360005) was downregulated 6.82-fold. This protein plays an important role in the transcriptional regulation of oxidative stress resistance and mainly regulates the transcription of genes involved in biosynthesis of amino acids, cell wall synthesis, and divalent metal ion transport (Mn^2+^, Zn^2+^, Mg^2+^)^52,53^. Inhibition of amino acid biosynthesis genes could be a consequence of decreased transcription of *soxR*. Expression of other genes involved in the oxidative stress response were also affected. The expression of superoxide dismutases (SOD)- (JMRS01_50032, JMRS01_860045) and cytochrome-c peroxidase (*ccp*; JMRS01_560029) -coding genes, involved in the direct elimination of toxic radicals, was promoted. On the other hand, glutathione biosynthesis-related genes and a range of genes involved in indirect oxidative stress resistance were inhibited. Expression of *rpoH* (JMRS01_260040) was downregulated with a FC of −2.98. In *E. coli*, RpoH regulates transcription of several heat shock proteins (HSP) and chaperones such as DnaK, DnaJ, GrpE, GroEL and GroES as well as proteases^54,55^. Concomitant with literature, expression of genes coding for those proteins decreased as well following *rpoH* repression in RSMG. In summary, the main direction of expression of stress response genes in axenic *C. testosteroni* in RSMG was downward, which was also observed in *L. reuteri* after growth in RSMG^18^. Decreasing expression of genes coding for chaperones could be a direct result of an energy deficit and a decrease in protein synthesis and, consequently, protein folding requirements.

**Table 2:** Differentially expressed genes involved in amino acid biosynthesis and degradation of axenic *Comamonas testosteroni* in randomized simulated microgravity (RSMG) growth conditions.

GeneID	Gene name	Description	Fold change
AMINO ACID BIOSYNTHESIS			
Multiple pathways			
JMRS01_60051	*asd*	Aspartate semialdehyde dehydrogenase	−31.78
Alanine, aspartate and glutamate			
JMRS01_340010	*ansB*	Putative L-asparaginase type II	−8.33
JMRS01_700160	*gltD*	Glutamate synthase small chain subunit	2.04
JMRS01_820040	*asdA*	Aspartate 4-decarboxylase	2.04
Glycine, serine and threonine			
JMRS01_510022	*itaE*	Threonine aldolase	2.53
JMRS01_550028	*dapdh*	Meso-dieaminopimelate D-dehydrogenase	−2.94
JMRS01_600042	*serA*	2-hydroxyacid dehydrogenase	−2.94
Cysteine and methionine			
JMRS01_360053	*metH*	Methionine synthase	−2.27
JMRS01_360273	*metXS*	Homoserine-O-succinyltransferase	2.39
JMRS01_360274	*metW*	Methionine biosynthesis protein	2.16
JMRS01_360400	*mtnN*	Adenosylhomocysteine nucleosidase	−2.94
JMRS01_530020	*metY*	O-acetyl-L-homoserine sulfhydrylase	−2.50
Valine, leucine and isoleucine			
JMRS01_60054	*leuD*	3-isopropylmalate dehydratase small subunit	−2.63
JMRS01_60055	*leuC*	3-isopropylmalate dehydratase large subunit	−3.22
JMRS01_210055	*leuA*	2-isopropylmalate synthase	−6.25
JMRS01_260037	*ilvD*	Putative dihydroxy-acid dehydratase	−2.27
JMRS01_360067	*ilvE*	Branched-chain amino acid aminotransferase	−2.86
JMRS01_560065	*leuD*	3-isopropylmalate dehydratase small subunit	−2.33
JMRS01_860066	*ilvD*	Dihydroxy-acid dehydratase	−2.08
Lysine			
JMRS01_260004	*lysA*	Diaminopimelate carboxylase	−3.85
JMRS01_380130	*lys1*	Saccharopine dehydrogenase	−2.78
JMRS01_410012	*dapA*	Dihydrodipicolinate synthase	−11.11
Arginine and proline			
JMRS01_150051	*argA*	Amino acid N-acetyltransferase	−3.13
JMRS01_360204	*argC*	N-acetyl-γ-glutamylphosphate reductase	−6.25
JMRS01_600032	*ocd*	Ornithine cyclodeaminase	−3.13
JMRS01_600033	*rocF*	Arginase	−2.78
Histidine			
JMRS01_700069	*hutG*	N-formylglutamate deformylase	2.38
JMRS01_700136	*hisA*	1-(5-phosphoribosyl)-5-[(5-phosphoribosylamino)methylideneamino] imidazole-4-carboxamide isomerase	−2.22
JMRS01_700145	*hisG*	ATP phosphoribosyltransferase	−2.70
Phenylalanine, tyrosine and tryptophan			
JMRS01_300045	NA	Chorismate mutase	−2.22
JMRS01_360043	*trpB*	Tryptophan synthase β-chain	−2.63
JMRS01_550016	*aroQ*	3-dehydroquinate dehydratase	−2.50
JMRS01_590020	*aroE*	Shikimate dehydrogenase	−3.33
AMINO ACID DEGRADATION			
Valine, leucine and isoleucine			
JMRS01_190026	*scoA*	Putative 3-oxoacid CoA transferase subunit A	−2.33
JMRS01_330069	*acsA*	Acetoacetyl-CoA synthetase	−2.13
JMRS01_380122	*mmsA*	Methylmalonate-semialdehyde dehydrogenase	−3.13
Histidine			
JMRS01_360012	*hutU*	Urocanase hydratase	−2.44
Tyrosine			
JMRS01_600104	*hpd*	4-hydroxyphenylpyruvate dioxygenase	−2.94
JMRS01_810062	*nagK*	Fumarylpyruvate hydrolase	−4.35
Tryptophan			
JMRS01_860320	*kynA*	Tryptophan 2,3-dioxygenase	−3.13

Nitrate:nitrite antiporter NarK-coding genes (JMRS01_360321, JMRS01_360322), involved in denitrification^56^, were upregulated with a FC of 4.59 and 11.16. In anoxic or micro-oxic conditions, *C. testosteroni* has been shown to increase transcription of *narK* and other denitrification genes^57^. Also, changes in denitrification gene expression were observed in an SMG study on *V. natriegens* and in *P. aeruginosa* after exposure to both SMG and spaceflight conditions and were linked to an anaerobic environment^10,11,58^. Due to limited mass transfer in RSMG, anoxic conditions may have been generated, thereby inducing denitrification for energy production without O_2_ as the final electron acceptor. Meanwhile, the presence of NO_3_^−^ in the SUSS medium could act as an additional inducer for denitrification gene expression. Moreover, cytochrome bo3 ubiquinol oxidase (*cyoABCD*; JMRS01_170046-49), usually expressed in O_2_-rich conditions^59^, was downregulated. In addition, while no aromatic compounds were available in the SUSS medium, genes of the hybrid aerobic degradation of benzoate pathway (*box*; benzoate oxidation) were underexpressed. This could implicate the activation of a broad transcriptional regulatory response to O_2_ limitation in RSMG in axenic *C. testosteroni* to diminish resource utilization for underused aerobic processes.

A wide array of genes involved in motility and biofilm formation were also differentially regulated in axenic *C. testosteroni* in RSMG. An overview of these genes is provided in Table 3. In the flagellar assembly gene cluster (JMRS01_1070011 – 55), 18 genes were differentially upregulated and expression of six more was significantly increased (*p* < 0.05). One pilus assembly gene cluster was downregulated, while two others were upregulated. Moreover, genes coding for capsular polysaccharide (CPS) export proteins were upregulated (*lipA*; JMRS01_380113, *lipB*; JMRS01_380114). CPSs play a role in biofilm formation but are also involved in protection against environmental threats and as virulence factors^60^. Finally, transcription of five genes coding for predicted diguanylate cyclases (DGCs) were upregulated while transcription of two was downregulated. Upregulation of flagellar assembly genes suggests increased motility and is also crucial for biofilm formation^61^. Pili also play a role in (non-flagellar) motility and in surface adhesion for biofilm formation^62^. Meanwhile, DGCs catalyze the formation of second messenger cyclic di-GMP (c-di-GMP) molecules, which, in high intracellular concentrations, repress motility and promote biofilm formation^63–66^. Finally, increased *narK* expression suggests NO_3_^−^ respiration, which enhances biofilm stability of *C. testosteroni* and increases DGC expression^57^. In addition, increased biofilm formation was described for the bacteria *Bacillus subtilis*^67^, *E. coli*^29^*, P. aeruginosa*^68,69^ and *S. maltophilia*^27^ and the yeast *C. albicans*^47^ during SMG and spaceflight. In concert with the findings in those studies, the gene expression profile of axenic *C. testosteroni* in RSMG also suggests a biofilm lifestyle.

**Table 3:** Differentially or significantly (*p* < 0.05) expressed genes related to motility of axenic *Comamonas testosteroni* in simulated microgravity.

GeneID	Gene name	Description	Fold change in RSMG	Fold change in LSMMG
Flagellar assembly				
JMRS01_50022	*flhD*	Master regulator, transcriptional activator	1.87	ND
JMRS01_810020	*fleN*	FleQ anti-activator protein	2.41	−1.57
JMRS01_1070016	*fliM*	C-ring protein	2.46	ND
JMRS01_1070015	*fliN*	C-ring protein	3.25	−1.97
JMRS01_1070014	*fliO/fliZ*	Export gate protein	3.58	−2.06
JMRS01_1070013	*fliP*	Export gate protein	3.46	−2.25
JMRS01_1070012	*fliQ*	Export gate protein	4.06	ND
JMRS01_1070011	*fliR*	Export gate protein	2.62	ND
JMRS01_1070024	N/A	Conserved protein of unknown function	2.03	ND
JMRS01_1070019	*fliJ*	ATPase complex	2.07	ND
JMRS01_1070017	*fliL*	Flagellum associated protein	1.91	ND
JMRS01_1070040	*flhB*	Export gate protein	1.80	ND
JMRS01_1070038	*flhF*	Polar landmark protein	2.35	ND
JMRS01_1070041	*flgZ*	c-di-GMP phosphodiesterase	2.22	−1.78
JMRS01_1070055	*flgM*	Negative regulator of flagellin synthesis, FliA anti-σ28 factor	−1.81	ND
JMRS01_1070036	*fliA*	RNA polymerase σ28-factor (class III activator)	2.85	−2.27
JMRS01_1070027	*cheY*	Chemotaxis protein CheY	−1.51	ND
JMRS01_1070029	*motA*	Motility protein A	1.83	ND
JMRS01_1070028	*motB*	Motility protein B	2.08	ND
JMRS01_1070031	*fliC*	Flagellin	2.22	ND
JMRS01_1070033	*fliD*	Flagellar hook-associated protein 2	1.64	−1.43
JMRS01_1070034	*fliS*	Flagellar biosynthesis protein	1.92	ND
JMRS01_1070035	*fliT*	Flagellar biosynthesis protein	1.65	−1.43
JMRS01_1070045	*flgI*	P-ring protein	2.40	ND
JMRS01_1070044	*flgJ*	Flagellar rod assembly protein	2.27	ND
JMRS01_1070043	*flgK*	Flagellar hook-associated protein 1	2.46	−1.95
JMRS01_1070042	*flgL*	Flagellar hook-associated protein 3	2.77	−2.03
Pilus formation				
JMRS01_160001	*cpaE2*	Pilus assembly protein CpaE	−2.48	ND
JMRS01_160007	N/A	Putative lipoprotein	−3.03	1.61
JMRS01_160008	N/A	Putative type II and III secretion system protein	−3.86	ND
JMRS01_160009	*cpaB*	Pilus assembly protein CpaB	−4.47	1.85
JMRS01_160010	*cpaA*	Prepilin peptidase	−2.73	ND
JMRS01_300024	*cpaB*	Pilus assembly protein CpaB	1.60	−1.61
JMRS01_300025	*cpaC*	Pilus assembly protein CpaC	1.93	−2.03
JMRS01_300026	*cpaE*	Pilus assembly protein CpaE	2.14	−2.21
JMRS01_300027	*cpaF*	Pilus assembly protein CpaF	2.04	−2.10
JMRS01_300028	*tadB*	Tight adherence protein B	2.50	−1.96
JMRS01_300029	*tadC*	Tight adherence protein C	2.13	−1.89
JMRS01_460006	*pilA*	Pilus assembly protein PilA	2.38	ND
JMRS01_460007	*cpaA*	Prepilin peptidase	1.89	−1.99
JMRS01_460008	*cpaB*	Pilus assembly protein CpaB	1.69	ND
JMRS01_460009	*cpaC*	Pilus assembly protein CpaC	1.38	ND
JMRS01_460010	*cpaE*	Pilus assembly protein CpaE	2.20	ND
JMRS01_460011	*cpaF*	Pilus assembly protein CpaF	1.57	ND
JMRS01_460012	*tadB*	Tight adherence protein B	1.87	ND
JMRS01_460013	*tadC*	Tight adherence protein C	1.55	ND
JMRS01_700091	*pilB*	Type IV pilus assembly ATPase PilB	2.11	ND
JMRS01_700092	*pilC*	Type IV pilus assembly protein PilC	1.85	ND
JMRS01_700093	*pilD*	Prepilin peptidase	2.55	ND

*ND* Not differentially expressed, *RSMG* Randomized simulated microgravity, *LSMMG* Low-shear simulated microgravity.

We also found indications of carbon limitation in the transcriptome of axenic *C. testosteroni* in RSMG. Several DEGs involved in the reversible initial gluconeogenesis or final steps of glycolysis were identified in RSMG. Downregulation of phosphoenolpyruvate (PEP) carboxykinase-coding (PEPCK) gene *pckG* (JMRS01_360114), responsible for the reversible rate-limiting step in the production of glucose precursors from oxaloacetate, and of acetyl-CoA synthetase gene *acsA* (JMRS01_330069) was observed. In combination with the decreased transcription of endpoint glycolysis genes, this indicates a reduced capacity for acetyl-CoA formation from carbon sources such as acetate and pyruvate for utilization in the TCA cycle. In this cycle, isocitrate lyase (*aceA*; JMRS01_10058) gene expression increased more than 4-fold and is an important step in the glyoxylate shunt (GS). Promotion of the GS has been observed in iron limitation^70^ and other stress conditions but also during carbon limitation^71^. Most importantly, the GS circumvents the two decarboxylation steps of the TCA and enables the use of 2-carbon compounds like acetate^72^. Moreover, diminished expression of specific TCA cycle genes (*sucA*; JMRS01_170029, *fumA*; JMRS01_320102, and *sdhCDA*; JMRS01_560039 – 41) prevents the production of the proteins responsible for the TCA cycle steps that are skipped by the GS. In the SUSS medium, acetate is the sole organic carbon source. Through the upregulation of the GS in RSMG and the downregulation of enzymes that required for underused TCA steps, *C. testosteroni* might have allocated more resources to the efficient utilization of acetate by the GS for biosynthesis of carbohydrate precursors. This could serve as a potential rescue pathway during periods of starvation due to limited mass transfer of acetate in RSMG.

Overall, the gene expression profile of axenic *C. testosteroni* in RSMG reflected nutrient- and oxygen-limiting conditions, which was also observed in other bacteria grown in SMG^10,11,13,44,73^. These observations included increased motility and biofilm formation, a shift in the carbon utilization metabolism, and a transition to an anaerobic lifestyle. It is clear that RSMG elicits a strong transcriptional response to nutrient depletion in *C. testosteroni*.

In contrast, no DEGs could be linked to energy conservation or nutritional deprivation in LSMMG in axenic *C. testosteroni*. DEGs involved in flagellar machinery were downregulated (Table 3). However, one *narK* gene (JMRS01_360321) was downregulated along with lowered transcription (FC = −2.90 to −4.66) of nitrate reductase-coding genes *narGHJI* (JMRS01_360323-326). As opposed to RSMG growth, *C. testosteroni* diminishes its NO_3_^−^ reduction capacities in LSMMG, thereby seemingly favoring an aerobic lifestyle. The combination of the higher final cell density and these observations counterintuitively seem to indicate a higher O_2_ availability and thus better mixing compared to NG. In Crabbe et al. dissolved oxygen (DO) in the medium was measured and no differences were observed between NG and LSMMG. However, a slightly decreased oxygen transfer rate was revealed in LSMMG, hampering *P. aeruginosa*’s ability to collect O_2_^10^. Axenic *C. testosteroni* might require less DO than *P. aeruginosa* to take up O_2_. Consequently, *C. testosteroni* could have enough O_2_ available in LSMMG, eliminating the necessity of resorting to anoxic processes to harvest energy.

#### RMSG and LSMMG transcriptomic responses are complementary for tripartite Comamonas testosteroni

**Fig. 4:**
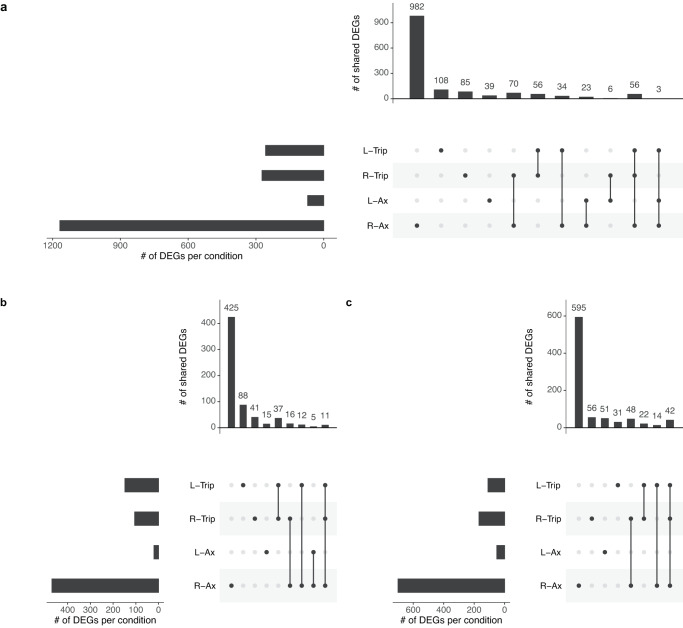
Overview of the extend of overlap in gene expression changes of axenic *Comamonas testosteroni* and in the tripartite community in simulated microgravity growth conditions. L-Trip and R-Trip refer to *C. testosteroni* in the tripartite community in low-shear modeled microgravity (LSMMG) and in randomized simulated microgravity (RSMG), respectively. R-Ax and L-Ax refer to axenically grown *C. testosteroni* in respectively RSMG and LSMMG. **a** represents all differentially expressed genes (DEGs). **b**, **c** represent the overlap of up- and downregulated genes between axenic and tripartite *C. testosteroni*, respectively.

*C. testosteroni* in a tripartite culture had 273 DEGs in RSMG conditions and 257 DEGs in LSMMG conditions (Fig. 4) (Supplementary Data 1). No DEGs in common were found across both SMG conditions in axenic and tripartite *C. testosteroni*. In RSMG, axenic and tripartite *C. testosteroni* shared 16 upregulated and 48 downregulated genes. In LSMMG, no common genes were found. In both tripartite culture SMG conditions, 37 and 22 common DEGs were respectively overexpressed or inhibited.

In tripartite *C. testosteroni*, upregulation of *narHJI* in RSMG and *narJ* and *narK* in LSMMG indicated increased denitrification capacities. This is in contrast to the downregulation of the genes of axenic *C. testosteroni* in LSMMG. The heightened expression of denitrification genes could be a direct effect of O_2_ competition with the nitrifiers in combination with SMG condition and the presence of NO_3_^−^ in the SUSS medium^4^. Expression of the *box*-pathway genes was also decreased in addition to an array of genes with roles in cell division, translation, purine and pyrimidine biosynthesis, and amino acid biosynthesis and degradation. These observations point at anoxic conditions and energy conservation^10,11,58^. Thereby, O_2_ transfer rates could be reduced to a level where *C. testosteroni* is also unable to gather enough O_2_ in this condition as opposed to its axenic LSMMG counterpart.

Expression of flagellar assembly master transcriptional regulator system *flhCD* was promoted up to 6.6-fold in LSMMG, as were several chemotaxis and aerotaxis receptor genes. Thus, a large part of the flagellar assembly gene cluster was promoted in LSMMG. On the other hand, only two genes were differentially expressed in RSMG, which highly contrasts with the axenic culture in RSMG. In both cases, DGC-coding genes were generally upregulated. In combination with heightened denitrification gene expression, it is plausible that SMG promotes biofilm formation in tripartite *C. testosteroni*^57^.

The metal ion homeostasis in tripartite *C. testosteroni* in RSMG and LSMMG was not altered to such a degree as in axenic *C. testosteroni* in RSMG. However, in contrast to axenic *C. testosteroni* in LSMMG, metal ion homeostasis of tripartite *C. testosteroni* was affected. Ferric siderophore uptake, Mn^2+^ export and arsenate efflux gene expression were increased in both conditions. As mentioned, increased expression of iron uptake genes implies iron limitation^51^, but in the tripartite culture, a lower response was noted in comparison to axenic *C. testosteroni* in RSMG.

The *rpoH* gene and the genes under its control, including several HSP-coding genes, *htpX*, and *htpG* were also downregulated in a tripartite setting in both scenarios. Gene expression of proteases that degrade damaged, truncated or misfolded proteins such as ClpP, HslV and MsrP and of proteins that confer oxidative stress resistance was also suppressed, similarly to axenic *C. testosteroni* in RSMG.

#### Differential gene expression of Nitrosomonas europaea in SMG

**Fig. 5:**
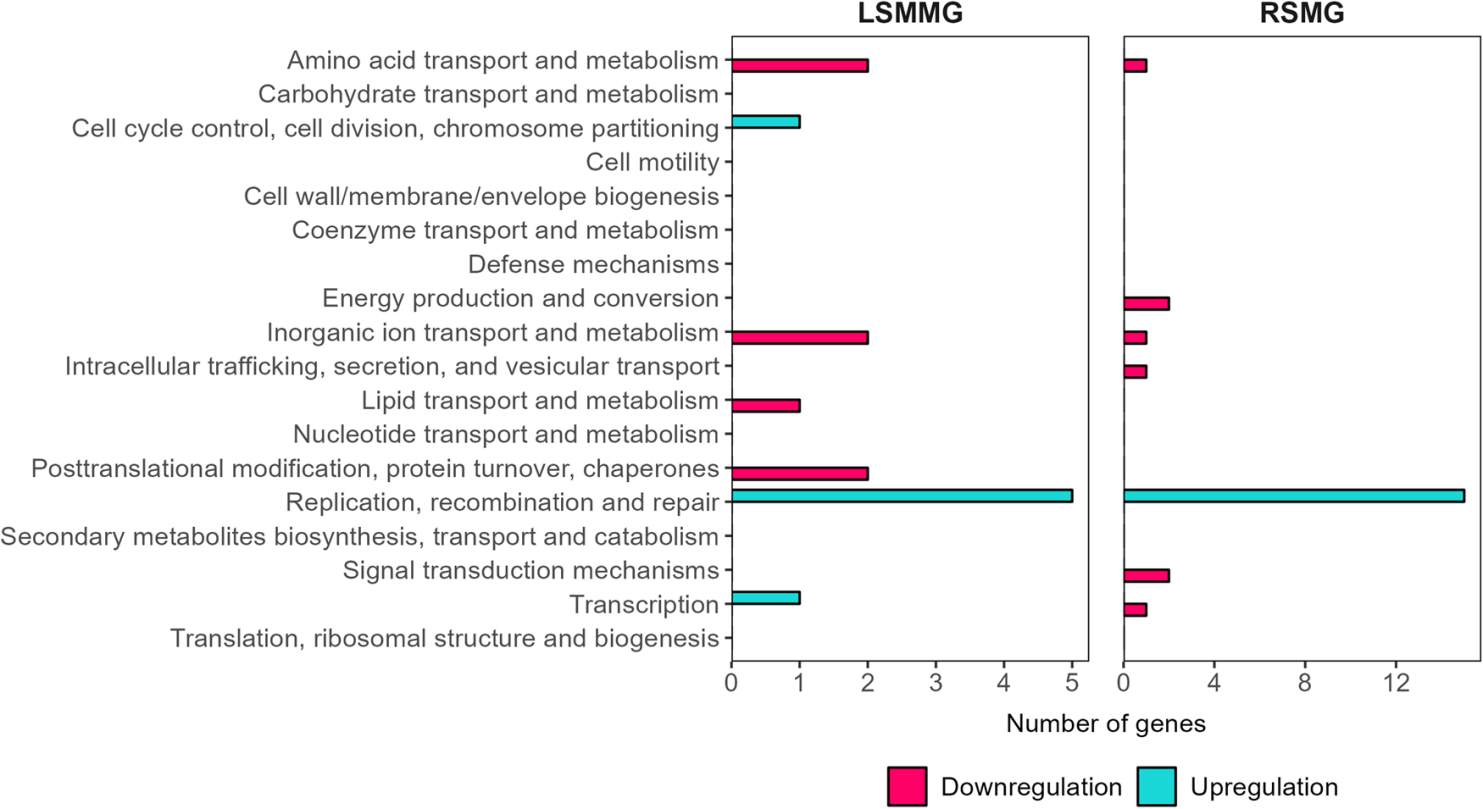
Differentially expressed genes per cluster of orthologues genes (COG) category of axenic *Nitrosomonas europaea* in simulated microgravity. Number of genes that are differentially regulated in each COG category in axenic *N. europaea* grown in low-shear modeled microgravity (LSMMG) and in randomized simulated microgravity (RSMG) conditions. COG categories ‘Function unknown’ and ‘General function prediction only’ were excluded.

For the studied aerobic ammonia oxidizer, we identified a total of 52 and 22 DEGs in the RSMG and LSMMG conditions, respectively, compared to NG (Supplementary Data 2). In the RSMG condition, transcript levels of 30 genes were increased while 22 genes were decreased. The transcriptomic response of *N. europaea* to SMG conditions was limited and only a handful of COGs were influenced (Fig. 5). In both cases, the ‘Replication, recombination and repair’ cluster was most affected. Respectively, three and 23 DEGs in RSMG and LSMMG were annotated ‘Function unknown’. ‘General function prediction only’ was the annotation of 5 DEGS is RSMG and 3 DEGs in LSMMG.

Most notably, members of the mercury import (*mer*) operon (NE0838–NE0842) were downregulated in both conditions. Transcription of all the operon’s genes was decreased in RSMG while in LSMMG, two genes (*merC/merP*; NE0840/41) were differentially downregulated and two more (*merA, merT*; NE0839, NE842) were close to the threshold of differential expression (FC < −1.97, *p* < 0.05). These proteins are responsible for mercury ion uptake and detoxification into the cytoplasm. Moreover, they were previously related to Cd^2+^ transport and are hypothesized to function as broad-range heavy metal transporters^74–76^. *SmbP* (NE2461), coding for a small metal-binding protein that removes toxic metal ions from the cell^77^, was also inhibited in RSMG. Reduction of heavy metal transport gene expression was observed in previous SMG experiments^11,44–47^, as mentioned, and in axenic and tripartite *C. testosteroni* in the current study. However, in contrast to RND transport systems, there is no proof of a functional role for the *mer*-operon in multidrug resistance. Its role is hence unclear and should be further explored before drawing any conclusions regarding its differential regulation in SMG.

In both SMG conditions, RuBisCO activase-coding genes *cbbO* and *cbbQ* (NE1918/19) were significantly downregulated. One subunit of the RuBisCO protein, CbbS (*cbbS*; NE1920) was also inhibited in LSMMG. Moreover, in RSMG, NH_4_^+^ oxidation seemed to be slightly affected, as reflected in the downregulation of *amoB2* (NE0943) and *amoA2* (NE2063). In LSMMG, no differential regulation of NH_4_^+^ oxidation genes was observed. *N. europaea* inhibits expression of all of the above genes in conditions of NH_4_^+^ and HCO_3_^−^ deprivation^78^, but increases expression of RuBisCO during carbon limitation only^79^. In the absence of NH_4_^+^, energy limitations are imposed on RuBisCO activity due to lack of NH_4_^+^ oxidation activity^79^. Moreover, RuBisCO expression has also been shown to decrease in O_2_-limiting conditions^80^. Hence, the inhibition of RuBisCO and ammonium monooxygenase gene expression could reflect a general nutrient deprivation profile for *N. europaea* in RSMG. In LSMMG, the effect is less pronounced but nonetheless present. Both SMG conditions could hence generate nutrient-depleted zones in axenic *N. europaea* cultures.

#### SMG has a small effect on the nitrification machinery of tripartite Nitrosomonas europaea

**Fig. 6:**
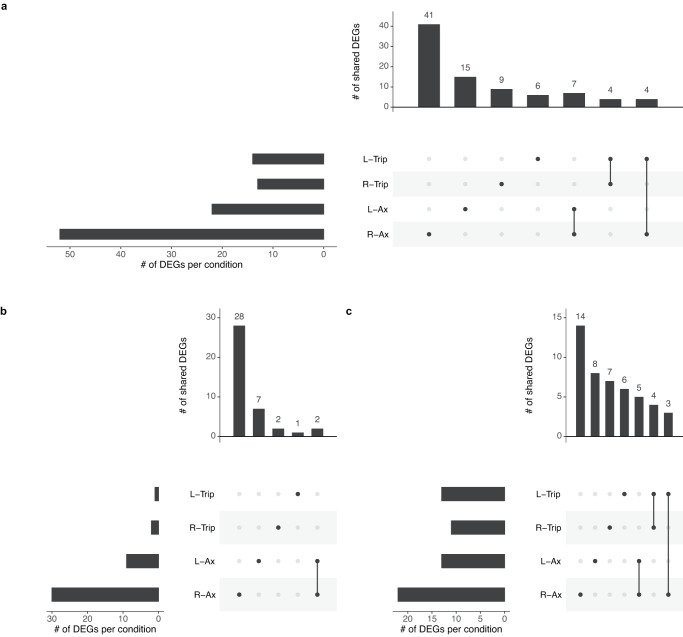
Overview of the extend of overlap in gene expression changes of axenic *N. europaea* and in the tripartite community in simulated microgravity growth conditions. L-Trip and R-Trip refer to *N. europaea* in the tripartite community in low-shear modeled microgravity (LSMMG) and randomized simulated microgravity (RSMG), respectively. R-Ax and L-Ax refer to axenically grown *N. europaea* in respectively RSMG and LSMMG. **a** represents all differentially expressed genes (DEGs). **b**, **c** represent the overlap of up- and downregulated genes between axenic and tripartite *N. europaea*, respectively.

In the tripartite community, respectively, 13 and 14 DEGs were identified in RSMG and LSMMG in the transcriptome of *N. europaea* (Supplementary Data 2). An overview of DEGs in common in the different SMG conditions of *N. europaea* grown axenically and in the tripartite community is provided in Fig. 6. No common DEG was identified across all four conditions.

Four genes were downregulated in both SMG conditions in the tripartite culture, while no common upregulated genes were found. Three of those DEGs are part of a significantly inhibited operon NE1538–NE1543 (*p* < 0.05). They code for two hypothetical proteins (NE1538, NE01539) and a TonB-dependent receptor protein (NE1540). Two of the remaining genes in the operon also code for hypothetical proteins (NE1541, NE1542) while NE1543 codes for a type I multicopper oxidase. Given that TonB-dependent receptor proteins and multicopper oxidases both have been implicated in iron acquisition, it is possible that this operon is involved in the tightly regulated iron homeostasis of *N. europaea*^81^. Additionally, in RSMG, expression of two putative *fecI* iron uptake σ-factors (NE1101, NE1207) was also inhibited. While not all iron-uptake related DEGs are identical in both conditions, together they indicate a reduced need for iron in SMG. This was also observed in *E. coli* in LSMMG and was suggested to prevent sulfur and cysteine consumption for Fe-S cluster assembly in starvation conditions^44^.

*NcgABC* (NE0925-7) expression was significantly decreased in RSMG (*p* < 0.05), but only *ncgA* displayed a FC lower than −2.00. These genes are clustered with nitrite reductase *nirK* (NE0924), required for efficient NH_4_^+^ oxidation and NO_2_^−^ resistance^82,83^. The genes are putatively involved in conferring NO_2_^−^ resistance by scavenging toxic NO molecules generated by NirK activity^83^. Significant inhibition of *ncgABC* expression suggests a decreased capacity to process NO_2_^−^ during NH_4_^+^ oxidation. In contrast to NO_2_^−^ accumulation in axenic *N. europaea* cultures, a part of the NO_2_^−^ might have been consumed by *N. winogradskyi* in the tripartite community. Consequently, NO_2_^−^ toxicity may have been decreased and *N. europaea* can dedicate less resources to NO_2_^−^ tolerance. In RSMG, this effect might be enhanced compared to NG, leading to a greater reduction of *ncgABC* expression. In turn, this suggests more efficient NO_2_^−^ consumption by *N. winogradskyi* in the tripartite community in RSMG as opposed to NG.

The *ncgABC-nirK* cluster was not differentially regulated in LSMMG compared to NG, indicating similar NO_2_^−^ consumption in these configurations. Also, *amoB2* (NE0943) expression was slightly increased (FC = 1.26). As opposed to axenically grown *N. europaea* in SMG, NH_4_^+^ availability seemed to be higher in LSMMG because of higher *amoB2* expression, increasing oxidation capacities compared to NG. However, the transcriptional differences to NG are very limited. A previous study already determined that N-species consumption and production were similar in LSMMG and NG in a tripartite community^4^, implicating that the gene expression profile observed here might not translate to a phenotypical level. However, to validate this, an N-species balance with the experimental setup used here should be conducted. In general, tripartite *N. europaea* was fairly unaffected by SMG conditions. This might be possible due to the vicinity and movement of *C. testosteroni*, thereby providing NH_4_^+^ for growth more efficiently than through diffusion only as would be the case in axenic *N. europaea*.

#### Nitrobacter winogradskyi’s altered gene expression in SMG

**Fig. 7:**
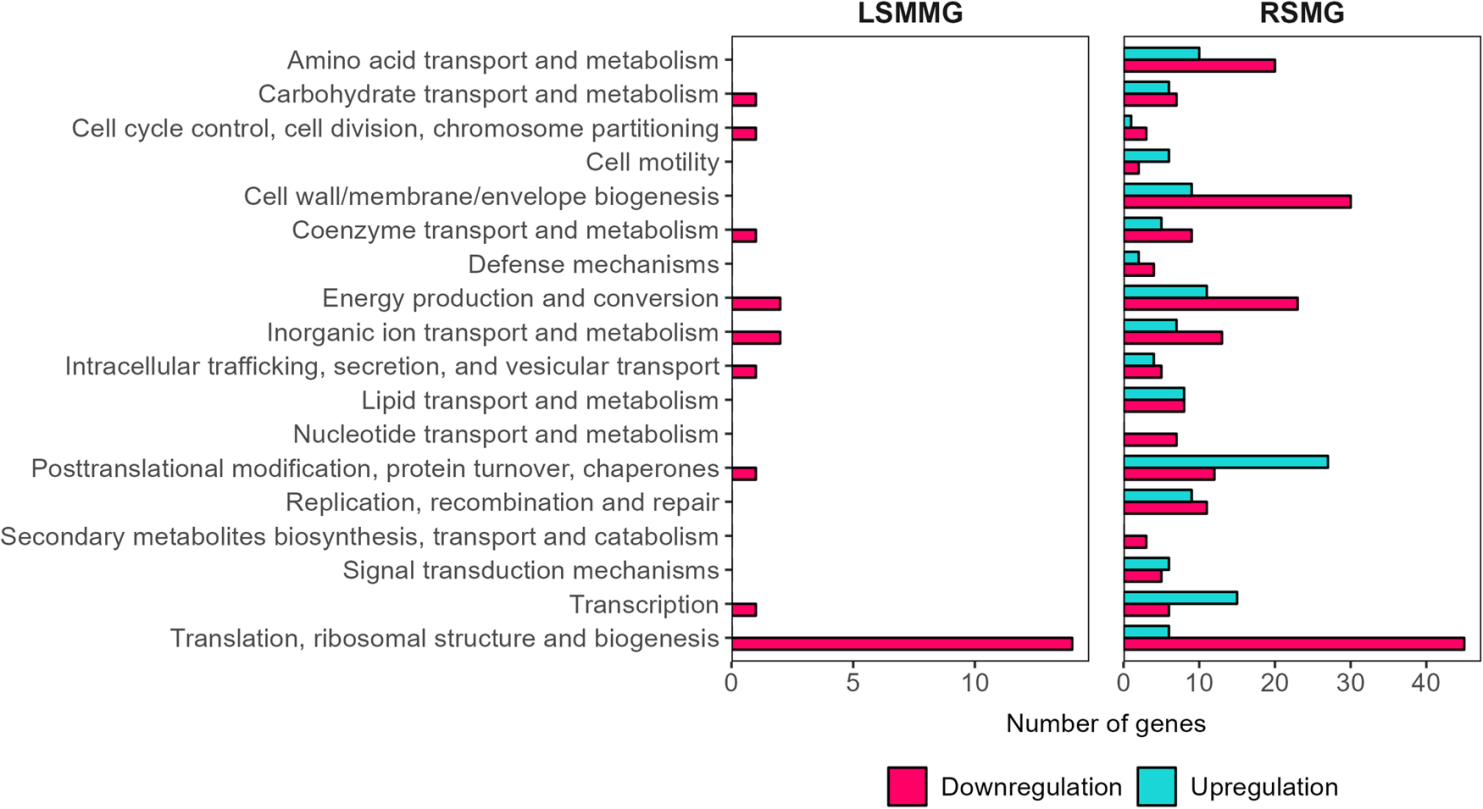
Differentially expressed genes per cluster of orthologues genes (COG) category of axenic *Nitrobacter winogradskyi* in simulated microgravity. Number of genes that are differentially regulated in each COG category in *N. winogradskyi* grown in low-shear modeled microgravity (LSMMG) and randomized simulated microgravity (RSMG) conditions. COG categories ‘Function unknown’ and ‘General function prediction only’ were excluded.

For the studied aerobic nitrite oxidizer, we identified 605 DEGs in RSMG and 40 DEGs in LSMMG compared to the NG control. In RSMG, 309 DEGs were upregulated and 296 were downregulated while in LSMMG, two DEGs were upregulated and 38 were downregulated (Supplementary Data 3). A COG analysis (Fig. 7) showed no upregulated genes annotated to a COG in LSMMG. In both cases, the ‘Translation, ribosomal structure and biogenesis’ COG was most represented. In RSMG, several other COGs stood out, including ‘Amino acid transport and metabolism’, ‘Cell wall/membrane/envelope biogenesis’, ‘Energy production and conversion’ and ‘Posttranslational modification, protein, turnover, chaperones’. Respectively 28 and 235 DEGs in RSMG and 16 and one DEGs in LSMMG were annotated to ‘Function unknown’ or ‘General function prediction only’.

#### Cell growth & proliferation of axenic Nitrobacter winogradskyi is hampered in SMG

In axenic *N. winogradskyi*, we observed a significant reduction in the transcript levels of various genes involved in DNA replication, transcriptional machinery, translation, and the cell cycle in RSMG (Table 4). The overall downregulation of transcription of these genes suggests a strong repression of proliferation-related processes in RSMG. In LSMMG, only genes involved in translation were downregulated. The decreased expression of cell growth and proliferation, and protein synthesis genes could indicate a preservation of energy. *N. winogradskyi* did display increased viability compared to the NG. In a previous study on *S. mutans*, translation-related gene expression was also downregulation without any observable changes in viability^39^. Therefore, reduced expression of proliferation-related genes does not necessarily impact viability. However, in the case of *N. winogradskyi*, increased viability could potentially be linked to a decelerated metabolism to limit their resource usage, resulting in a reduced cellular turnover. In addition, RuBisCO- and carboxysome-related genes (Nwi_1975/76, Nwi_1980–1987) were strongly downregulated with FCs ranging from −4.99 to −34.78 in RSMG and from −2.15 to −4.47 in LSMMG. Gene expression of master regulator *cbbR1* (Nwi_1988) was also decreased in both conditions. Meanwhile, in RSMG only, master regulator *cbbR2* (Nwi_2930) was upregulated. Overexpression of *cbbR2* in RSMG could favor transcription of the second, standalone RuBisCO copy (*cbbS2/L2*; Nwi_2928/29) in the *N. winogradskyi* genome. This RuBisCO copy is not associated with a carboxysome structure^84^ and was not differentially expressed in both conditions. However, to maintain carbon fixation in the energy conserving condition to which *N. winogradskyi* seems to transition in SMG, this RuBisCO copy could be adequate to capture the necessary amount of CO_2_ without association with a carboxysome for cell maintenance and survival. This could be a consequence of low O_2_ availability in SMG, reducing competition for RuBisCO binding and allowing more efficient CO_2_ fixation^80^.

**Table 4:** Proliferation-related differentially expressed genes in axenic *Nitrobacter winogradskyi* in simulated microgravity.

Gene ID	Gene name	Description	Fold change in RSMG	Fold change in LSMMG
Replication				
Nwi_0001	*dnaA*	Chromosomal replication initiation protein	−2.96	ND
Nwi_0002	*dnaN*	Polymerase III subunit β	−2.08	ND
Nwi_0105	*dnaQ*	Polymerase III subunit ε	−2.27	ND
Nwi_1062	*ligA*	DNA ligase	−2.09	ND
Nwi_2603	*ccrM*	Cell cycle-regulated DNA methyltransferase	−3.15	ND
Transcription				
Nwi_1351	*rpoC*	DNA-directed RNA-polymerase subunit β’	−2.64	ND
Translation				
Nwi_0065	*pheT*	Phenylalanine-tRNA ligase subunit β	−2.91	ND
Nwi_0066	*pheS*	Phenylalanine-tRNA ligase subunit α	−2.69	ND
Nwi_0446	*rsfS*	Ribosomal silencing factor	2.20	ND
Nwi_1346	*rplK*	50 S ribosomal protein L11	−2.33	ND
Nwi_1348	*rplJ*	50 S ribosomal protein L1	−2.27	ND
Nwi_1359	*rpsL*	30 S ribosomal protein S12	−3.16	ND
Nwi_1360	*rpsG*	30 S ribosomal protein S7	−5.39	ND
Nwi_1361	*fusA*	Elongation factor G	−5.11	ND
Nwi_1362	*tuf*	Elongation factor Tu	−5.34	ND
Nwi_1363	*rpsJ*	30 S ribosomal protein S10	−8.71	−1.99
Nwi_1364	*rplC*	50 S ribosomal protein L3	−4.85	ND
Nwi_1365	*rplD*	50 S ribosomal protein L4	−5.55	−2.13
Nwi_1366	*rplW*	50 S ribosomal protein L23	−8.96	−2.23
Nwi_1367	*rplB*	50 S ribosomal protein L2	−5.08	ND
Nwi_1368	*rpsS*	30 S ribosomal protein S19	−6.71	−3.09
Nwi_1369	*rplV*	50 S ribosomal protein L22	−9.41	−2.23
Nwi_1370	*rpsC*	30 S ribosomal protein S3	−6.20	ND
Nwi_1371	*rplP*	50 S ribosomal protein L16	−7.22	ND
Nwi_1372	*rpmC*	50 S ribosomal protein L29	−6.55	−3.01
Nwi_1373	*rpsQ*	30 S ribosomal protein S17	−5.80	−3.45
Nwi_1374	*rplN*	50 S ribosomal protein L14	−8.13	−3.27
Nwi_1375	*rplX*	50 S ribosomal protein L24	−10.18	−3.53
Nwi_1376	*rplE*	50 S ribosomal protein L5	−6.87	−2.87
Nwi_1377	*rpsN*	30 S ribosomal protein S14	−5.02	ND
Nwi_1378	*rpsH*	30 S ribosomal protein S8	−6.43	−2.67
Nwi_1379	*rplF*	50 S ribosomal protein L6	−4.65	ND
Nwi_1380	*rplR*	50 S ribosomal protein L18	−4.05	−2.48
Nwi_1381	*rpsE*	30 S ribosomal protein S5	−6.29	−2.30
Nwi_1382	*rpmD*	50 S ribosomal protein L30	−5.23	−3.09
Nwi_1383	*rplO*	50 S ribosomal protein L15	−4.78	−2.20
Nwi_1384	*secY*	preprotein translocase subunit SecY	−3.79	−2.05
Nwi_1588	*aspS*	Aspartate-tRNA ligase	−2.17	ND
Nwi_1776	*serS*	Seryl-tRNA synthetase	−2.07	ND
Nwi_1858	*tsf*	Elongation factor Ts	−2.93	ND
Nwi_1859	*rpsB*	30 S ribosomal protein S2	−2.72	ND
Nwi_1918	*rnc*	Ribonuclease 3	−2.35	ND
Nwi_2586	*glyS*	Glycyl-tRNA synthetase	−4.40	ND
Cell cycle				
Nwi_1047	*murF*	UDP-N-acetylmuramoyl-tripeptide--D-alanyl-D-alanine ligase	−2.37	ND
Nwi_1051	*murG*	UDP-N-acetylglucosamine--N-acetylmuramyl-(pentapeptide) pyrophosphoryl-undecaprenol N-acetylglucosamine transferase	−2.87	ND
Nwi_1056	*ftsQ*	Cell division protein FtsQ	−2.79	ND
Nwi_1057	*ftsA*	Cell division protein FtsA	−2.42	ND
Nwi_1803	*scpB*	Condensin subunit ScpB	−2.17	ND
Nwi_1804	*scpA*	Condensin subunit ScpA	−2.36	ND
Nwi_2717	*tolB*	Tol-Pal system protein TolB	−2.39	ND

*ND* Not differentially expressed, *RSMG* Randomized simulated microgravity, *LSMMG* Low-shear modeled microgravity.

Several genes in the central carbon metabolism were also upregulated in RSMG. PEPCK (*pckA*; Nwi_0350), acetyl-CoA synthetase (*acsA*, Nwi_0467), and succinate dehydrogenase (*sdhCD*; Nwi_2789/9) expression was increased. Acetate stemming from fatty acid (FA) β-oxidation is activated by AcsA, increasing carbon availability to enter the TCA cycle. Meanwhile, FA catabolism produces additional energy during energy-limiting conditions. The upregulation of PEPCK may be needed to enhance oxaloacetate production from PEP, additionally increasing flux towards oxaloacetate. This transcriptomic profile of axenic *N. winogradskyi* in RMSG is indicative of anaplerosis to reinforce acetate-CoA consumption. Hence, *N. winogradskyi* may be using carbon from FA degradation to maintain its central metabolism in the TCA cycle during starvation conditions.

Expression of a nitrite oxidoreductase (NXR) subunit β gene (*nxrB*; Nwi_0965) was increased 7.11-fold in RSMG, which is implicated in NO_2_^−^ oxidation but also NO_3_^−^ reduction. Given that the transcriptomic profile of axenic *N. winogradskyi* in RSMG is indicative of nutrient limitation, there are two possibilities for the upregulation of NXR. For one, NO_2_^−^ might be present in limiting quantities, resulting in increased transcription of NXR for energy production. On the other hand, O_2_ could be a limiting factor, as suggested by the expression pattern of RuBisCO. In this case, NXR upregulation would indicate an anoxic environment^85^ and is necessary to increase denitrification activities of *N. winogradskyi*. Furthermore, the presence of NO_3_^−^ in the SUSS medium could also be an additional trigger for *nxr* upregulation in this case. In future work, it is thus highly recommended to assess the evolution of N-species in the medium, which could bolster the validity of one of the hypotheses.

For both SMG conditions, the transcriptomic profile is indicative of a conservation of energy and a limitation of translation- and transcription-related processes. In RSMG, this behavior is more accentuated than in LSMMG.

#### Axenic Nitrobacter winogradskyi increases oxidative stress resistance in RSMG

As opposed to *C. testosteroni* in SMG, expression of *rpoH* (Nwi_2430) was increased 2.20 fold. As a possible result, RpoH increased gene expression of stress response chaperone systems DnaK, DnaJ, GroEL/GroES, proteases Lon, and FtsH (Nwi_2710). Gene transcripts of several HSPs were also present in elevated quantities. Furthermore, ROS detoxification enzymes catalase KatG (Nwi_0030), thioredoxin TrxA (Nwi_0051), alkylhydroperoxidase AhpD (Nwi_1458) and glutathione S-transferase (Nwi_2981) gene expression was increased in RSMG, while other oxidative stress response protein-coding genes were downregulated. Genes with a role in DNA repair, stabilization and protection were upregulated. All of the aforementioned genes are related to stress responses, mainly in response to increased ROS levels. Hence, we hypothesize that *N. winogradskyi* is increasing its resistance to oxidative stress during growth in RSMG. No stress response genes were differentially regulated in LSMMG.

#### The transcriptomic profile of tripartite Nitrobacter winogradskyi is indicative of an anoxic environment

**Fig. 8:**
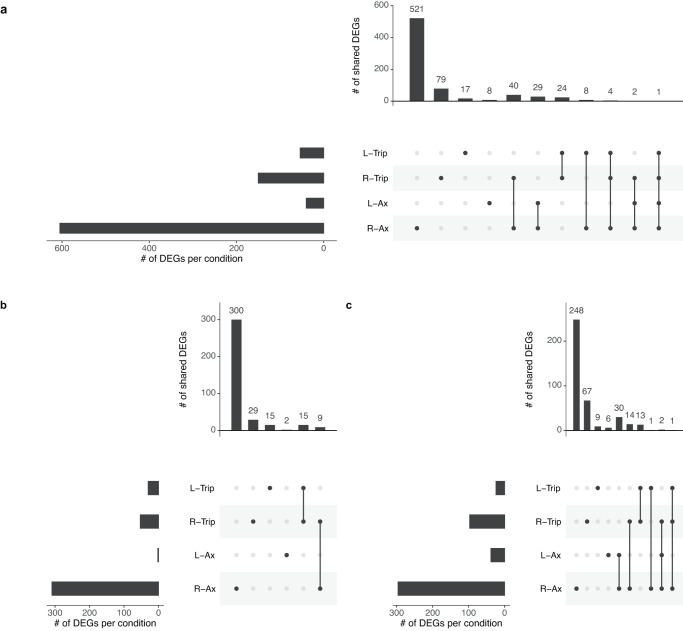
Overview of the extend of overlap in gene expression changes of axenic *Nitrobacter winogradskyi* and in the tripartite community in simulated microgravity growth conditions. L-Trip and R-Trip refer to *N. winogradskyi* in the tripartite community in low-shear modeled microgravity (LSMMG) and in randomized simulated microgravity (RSMG), respectively. R-Ax and L-Ax refer to axenically grown *N. winogradskyi* in respectively RSMG and LSMMG. **a** represents all differentially expressed genes (DEGs). **b**, **c** represent the overlap of up- and downregulated genes between axenic and tripartite *N. winogradskyi*, respectively.

Respectively 150 and 54 DEGs were identified in tripartite *N. winogradskyi* in RSMG and LSMMG (Fig. 8) (Supplementary Data 3). The bacteria in the synthetic community had 17 distinct DEGs in common with the axenic *N. winogradskyi* in RSMG and none in common in LSMMG. No DEG was exclusively up- or -downregulated across all conditions.

Expression of four HSP-20 coding genes and *groL1*, *groES* in RSMG, and one HSP-20 coding gene and *groL2* LSMMG were repressed, indicating a reduced capacity for protein folding, assembly, transport and degradation in both SMG conditions compared to NG. In RSMG, upregulation of a Trx-like protein (Nwi_0716), DegP-like endoprotease (Nwi_1195) and Fe-S repair gene cluster (*sufBCDS*; Nwi_1661-64) indicated a requirement to repair or degrade damaged proteins. Meanwhile, two genes (Nwi_2605, Nwi_1513) involved in DNA base excision repair were downregulated.

The transcriptomic profile of tripartite *N. winogradskyi* in RSMG also shows several signs of nutritional stress. For one, expression of ATP synthase subunit α (*atpA*; Nwi_0430), *cbbL1*, and *cbbR1* was inhibited, possibly due to energy conservation and carbon shortages. The differential expression of some other genes also suggests anoxic conditions. Peptidase T-coding gene (*pepT*; Nwi_1893) was overexpressed and is usually induced in an anaerobic growth setting with the goal of catabolizing amino acids to meet the energy demands of the cell^86^. Also, NO_3_^−^ import was increased in both SMG conditions through the upregulation of *nark* (Nwi_0779) to putatively increase denitrification capabilities. Expression of *sdhCD* was also increased, which, like in axenic *N. winogradskyi*, could be an indicator of a metabolic flux towards oxaloacetate and anaplerosis during starvation. No differentially expressed NXR genes were discovered. Finally, DEGs involved in amino acid biosynthesis were predominantly downregulated and glycine degradation was inhibited. Interestingly, integration host factor (IHF) subunit β (*ihfB*; Nwi_0058) was upregulated in both conditions. IHF regulates the expression of genes required for the physiological transition from the exponential to the stationary growth phase^87^, which usually occurs when nutrients are depleted. Like its axenic counterpart, tripartite *N. winogradskyi* also shows signs of survival in a nutrient-depleted environment in SMG.

#### Implications and outlook

Across all bacterial strains and culture configurations, only the axenic *C. testosteroni* strain in LSMMG did not express a starvation-related response in its gene expression profile. On the contrary, it seemed to experience a more aerobic lifestyle compared to NG. However, the gene expression differences were very limited compared to NG with only 71 DEGs identified. In all other bacteria, the gene expression profiles suggest that O_2_, carbon, nutrients and electron donors such as acetate, NH_4_^+^ and NO_2_^−^, depending on the bacterial strain, were constraining factors. Especially O_2_ availability is limiting, as suggested by the upregulation of the denitrification machinery across the strains (possibly also linked to the presence of NO_3_^−^ in the SUSS medium). The variations in the number of DEGs suggest differences in responses depending on the SMG condition and the presence of other bacterial strains in the culture. These aspects affect how the N-cycle bacteria adapt their gene expression. Nonetheless, the similar functionalities in the gene expression patterns imply that the fluid dynamics in SMG negatively influences the mass transfer of nutrients and diffusion of O_2_ in liquid medium.

In the scope of nitrogen recovery in space, one could infer that nitrification efficiency will be affected in a microgravity environment. Increased denitrification-related gene expression, as was observed on multiple occasions, could have an impact on the nitrification efficiency and the recovery percentages. This hypothesis may be confirmed in future work by assessing phenotypical characteristics of the bacteria and an N-species balance. In the context of RLSS design, denitrification should be avoided in a system like MELiSSA to prevent nitrogen losses. In alternative LSS scenarios, biological nitrogen fixation could be envisaged for food production, or nitrogen gas can be valorized as inert gas compensating for leaks in the artificial cabin or habitat atmosphere. Nonetheless, in the conventional route with nitrate-based fertilizer production, the space microgravity environment could pose a major challenge for efficient nitrogen recovery from waste streams.

A spaceflight experiment called ‘Urine Nitrification in Space’ (URINIS) will be performed to assess the effects of the real spaceflight environment on nitrification and will prove valuable in assessing the complete impact of spaceflight (microgravity, ionizing radiation, etc.) on N-cycle bacteria. However, if the nutritional-deprivation-induced transcriptomic responses observed in this work are also observed during the spaceflight, optimization of mixing within the nitrifying bioreactor should be carefully considered. By providing a homogenously mixed environment, the impact of minimized fluid dynamics and associated diffusional limitations in microgravity may be mitigated.

Transcriptomic responses of N-cycle bacteria highlighted that the strains are subjected to limited mass transfer due to strongly reduced fluid dynamics in SMG. Almost all strains experienced some form of nutrient and O_2_ depletion. In this context, RSMG almost exclusively elicited a stronger response than LSMMG compared to NG. Despite apparent nutritional deprivation in the gene expression profile in response to SMG, urea hydrolysis and nitrification genes were almost never affected. Only in *N. europaea* in the tripartite culture and axenic *N. winogradskyi*, a limited effect was noticed on these genes. Conversely, denitrification gene expression was upregulated in *C. testosteroni* and *N. winogradskyi*. In the former, biofilm formation was also promoted. From these results, it is possible that the impact on nitrification efficiency could be substantial in microgravity. Conclusive insights into the impact of spaceflights on nitrification in MELiSSA require spaceflight experiments conducted in batch and bioreactor configurations.

## Methods

### Bacterial cultivation

Bacterial strains were grown in synthetic urine salt solution (SUSS) medium based on the medium described in Ilgrande et al.^4^ which was composed of 0.15 g L^−1^ NaNO_3_, 1.564 g L^−1^ KH_2_PO_4_, 0.49 g L^−1^ MgSO_4_ • 7H_2_O, 0.04 g L^−1^ CaCl_2_ • 2H_2_O, 0.0014 g L^−1^ FeSO_4_ • 7H_2_O, 5.2 g L^−1^ NaCl, 2 g L^−1^ K_2_HPO_4_, 2.5 g L^−1^ KHCO_3_, 3.2 g L^−1^ Na_2_SO_4_ • 10H_2_O, 37.85 g L^−1^ EPPS. 0.5 g L^−1^ Na-acetate and 1.07 g L^−1^ urea were added to SUSS medium for *C. testosteroni* I2 and brought to a pH of 7. The same is true for the tripartite culture, but pH was brought to 7.8. For *N. europaea* ATCC 19718, 2.36 g L^−1^ (NH_4_)_2_SO_4_ was added to the SUSS medium and pH was adjusted to 7.8, whereas 2.46 g L^−1^ NaNO_2_ was added for *N. winogradskyi* Nb-255 and pH was adjusted to 7.5.

*C. testosteroni* was grown in Lennox L Broth Base (LB) (ThermoFisher Scientific) at 30 °C in the dark on an orbital shaker at 120 rpm in ventilated cell culture flasks. After 2 days of growth, the culture was transferred 5% (v/v) to fresh SUSS medium and grown for 3 days in ventilated cell culture flasks. The culture was transferred 5% (v/v) to 95 mL of fresh SUSS medium in a PL-70 cell culture bag and grown in SMG conditions for 3 days. Axenic strains of *N. europaea* and *N. winogradskyi* were grown in SUSS medium in ventilated cell culture flasks at 30°C in the dark on an orbital shaker set at 120 rpm for 5 days.

### Simulation of microgravity

The RPM and RCCS were used to grow the bacterial strains in SMG conditions. *C. testosteroni* and the tripartite culture were cultured in PermaLife^TM^ PL-70 cell culture bags (Origen Biomedical). *N. europaea* and *N. winogradskyi* were grown in Synthecon RCCS bioreactors (Synthecon, Inc.). The bacterial cultures in their respective containers were mounted to the RPM (RSMG), to the RCCS rotator with its axis perpendicular to the gravity vector (LSMMG) or to the RCCS rotator with the axis parallel to the gravity vector (NG). For the PL-70 cell culture bags, custom in-house designed 3D holders were used to mount to bags to the respective microgravity simulators. The RCCS rotator was rotated at 25 rpm. The RPM was operated as a random walk three-dimensional clinostat as described in Mastroleo et al.^16^.

*C. testosteroni* was transferred 5% (v/v) to 90 mL of fresh SUSS medium in a PL-70 cell culture bag. *N. europaea* and *N. winogradskyi* were inoculated 10% (v/v) in 50 mL RCCS bioreactors. The tripartite culture was assembled with the separate axenic cultures of *C. testosteroni*, *N. europaea* and *N. winogradskyi*. 1/3^rd^ of every strain was combined in a 10% (v/v) transfer to 90 mL fresh SUSS medium and added to a PL-70 cell culture bag. All cultures were grown in SMG conditions for respectively 3, 5, 5 and 20 days in the dark at 30 °C.

### Assessment of fluid-mixing in PL-70 cell culture bags in RSMG, LSMMG and NG

PL-70 cell culture bags were filled with 100 mL of distilled water and air bubbles were meticulously removed. The bags were mounted to the RCCS and RPM devices and rotated for 5 min before injection with 600 µL of a 0.03% crystal violet solution with a 1 mL syringe. The dispersion of the dye was monitored with pictures using a smartphone camera.

### OD_600_ measurements

The optical density, to measure bacterial growth, was determined on 500 µL aliquots with a NanoColor UV/Vis II spectrophotometer (Machery-Nagel) at wavelength *λ* = 600 nm (OD_600_).

### LIVE/DEAD analysis

LIVE/DEAD analysis was performed using flow cytometry. The samples were diluted and stained with nucleic acid stains. A combination of SYBR Green I (SG) combined with propidium iodide (PI) (SGPI, 100x concentrate SG, Invitrogen, and 50 × 20 mM PI, Invitrogen, in 0.22 µm-filtered dimethyl sulfoxide) was used for the analysis. The samples were stained by adding 10 µL mL^−1^ of SGPI solution followed by incubation for 20 min in the dark at 37 °C. Three technical replicates were prepared per biological replicate. A BD Accuri C6 flow cytometer (BD Biosciences) was used for flow cytometric analysis using the 488 and 640 nm laser for excitation of the fluorescent dyes.

### Relative abundance of strains in the tripartite community

DNA from 10 mL of tripartite culture was extracted using the QiAMP DNA Mini kit (Qiagen) according to the manufacturer’s instructions. The relative abundance of *C. testosteroni*, *N. europaea* and *N. winogradskyi* in the tripartite culture was assessed with real-time quantitative PCR (qPCR) using the ΔΔC_T_ method^88^. Universal 16 S rRNA primers 910 FW and 1141 RV (AGCGGTGGATGATGTGGATTAA, TTGTCACCGGCAGTCTCTCTAG) and species-specific primers *ureA* (AGCGCCTTTGTGATGGAA, GATCTGGATGTCGGGAATCATC), *amoA* (ACACCCGAGTATGTTCGTCA, TGCGATGTACGATACGACCT), and *nxrA* (GAGATGCAGCAGACCGACTA, GGCTGTAGACGTACCACGAA) for *C. testosteroni*, *N. europaea* and *N. winogradskyi*, respectively, were used. Primers for *ureA* were designed for this experiment, while *amoA* and *nxrA* primers were used according to Perez et al., 2015^40^. The qPCR cycling parameters were 5 min at 95 °C followed by 35 cycles of 15 sec at 95 °C and 1 min at 65 °C. The program was executed on the real-time PCR cycler RotorGene Q (Qiagen). 25 µL of qPCR mixture was used with QuantiNova SYBR Green RT PCR (Qiagen), 300 nM of FW and RV primers and 5 ng of DNA. qPCR of the genomic DNA standards, non-template controls and samples were performed in technical triplicate. Since *N. europaea* and *N. winogradskyi* contain 2 copies of *amoA* and *nxrA* respectively, the calculated fold change (2^−ΔΔCT^) for these species was divided by 2.

### RNA extraction

10 mL of bacterial culture samples for *C. testosteroni* and the nitrifiers were pelleted by centrifugation at 14,000 g for 5 min. RNA was isolated according to an optimized protocol for low-biomass bacterial samples^89^ as described in Verbeelen et al.^90^. RNA samples with a RIN-value above or equal to 8 were accepted for sequencing. RNA-Seq was performed on biological triplicates.

### RNA sequencing

RNA sequencing procedure was outsourced to BaseClear B.V. (Leiden, The Netherlands). Here, rRNA was first depleted using the Illumina Ribo-Zero Plus kit (Illumina). Paired-end sequence reads were generated using the Illumina NovaSeq 6000 system (Illumina). The Illumina TruSeq Stranded Total RNA kit was used to construct the library. FASTQ read sequence files were generated using bcl2fastq version 2.20 (Illumina). Initial quality assessment was based on data passing the Illumina Chastity filtering. Subsequently, reads containing PhiX control signal were removed using an in-house filtering protocol. In addition, reads containing (partial) adapters were clipped (up to a minimum read length of 50 bp). The second quality assessment was based on the remaining reads using the FASTQC quality control tool version 0.11.8 (Brabraham Bioinformatics).

### RNA-Seq data analysis

Paired-end mRNA reads were mapped with subread for R (version 2.0.1)^91^ to the reference genome of the strain (*C. testosteroni* I2; NCBI accession number CP067086.1, *N. europaea* ATCC19718; NCBI accession number AL954747.1, *N. winogradskyi* Nb-255; NCBI accession number CP000115.1). For the tripartite community, the 3 genomes were combined to perform the mapping process. Gene expression quantification was performed with the featureCounts function^92^ from the subread package with the latest genome annotations available for *C. testosteroni* I2, *N. europaea* ATCC19718 and *N. winogradskyi* Nb-255 obtained from the MaGe platform. Differential gene expression was calculated using the edgeR (version 3.34.1)^93^ and limma (version 3.48.3)^94^ packages. Lowly expressed genes were filtered out using the filterbyExpr function of the edgeR package. Thresholds for differential gene expression (DGE) were a *p*-value < 0.05 and −1 ≥ log_2_ FC ≥ 1 ( | FC | ≥ 2).

### Statistical analysis

All experiments were performed in biological triplicate or quadruplicate, where stated. One-way Analysis of Variance (ANOVA) and post-hoc Tukey tests were performed to identify significant differences in endpoint OD_600_ measurements and LIVE/DEAD ratios of the bacterial cultures, *p* < 0.05 was considered statistically significant.

### Data visualization

Endpoint OD_600_-values, LIVE/DEAD ratios and relative abundancies of the tripartite culture were visualized using Graphpad Prism version 9.0.0 for Windows (GraphPad Software). COG barplots were constructed with the ggplot2 package for R (version 3.4.2). The UpSetR package for R (version 1.4.0)^95^ was used to build gene overlap plots for DEGs across different growth conditions.

### Supplementary information

Supplementary Data 1

Supplementary Data 2

Supplementary Data 3

Video of bacterial culture floc in LSSMG.

## Data Availability

The datasets generated and analyzed during the current study are available within the NCBI Sequence Read Archive (SRA) using the accession PRJNA881961.
